# Pharmacokinetics, safety, and tolerability of the 2‐ and 3‐direct‐acting antiviral combination of AL‐335, odalasvir, and simeprevir in healthy subjects

**DOI:** 10.1002/prp2.395

**Published:** 2018-04-30

**Authors:** Thomas N. Kakuda, Matthew W. McClure, Christopher Westland, Jennifer Vuong, Marie‐Claude Homery, Gwendoline Poizat, Laure Viguerie, Caroline Denot, Alain Patat, Qingling Zhang, James Hui, David Apelian, David B. Smith, Sushmita M. Chanda, John Fry

**Affiliations:** ^1^ Alios BioPharma, Inc., part of the Janssen Pharmaceutical companies South San Francisco CA USA; ^2^ Biotrial Rennes France; ^3^ Achillion Pharmaceuticals, Inc., New Haven CT USA

**Keywords:** AL‐335, drug safety, drug‐drug interactions, hepatitis C virus, odalasvir, pharmacokinetics, simeprevir

## Abstract

This Phase I, open‐label, two‐group, fixed‐sequence study evaluated the pharmacokinetics and safety of AL‐335, odalasvir, and simeprevir in healthy subjects. Group 1 (n* *=* *16) received AL‐335 800 mg once daily (QD) (days 1‐3, 11‐13, and 21‐23), simeprevir 150 mg QD (days 4‐23), and odalasvir 150 mg (day 14) followed by 50 mg QD (days 15‐23). Group 2 (n* *=* *16) received the same AL‐335 regimen as in Group 1 plus odalasvir 150 mg (day 4) followed by 50 mg QD (days 5‐23) and simeprevir 150 mg QD (days 14‐23). Blood samples were collected to determine plasma concentrations of AL‐335 (prodrug) and its metabolites, ALS‐022399 (monophosphate precursor) and ALS‐022227 (parent nucleoside), odalasvir, and simeprevir. Thirty‐two subjects were enrolled. Odalasvir and simeprevir given alone, or in combination, increased AL‐335 area under plasma concentration‐time curve over 24 hours (AUC_0‐24 h_) 3‐, 4‐, and 7‐ to 8‐fold, respectively; ALS‐022399 AUC_0‐24 h_ increased 2‐, 2‐, and 3‐fold, respectively. Simeprevir had no effect on ALS‐022227 AUC_0‐24 h_, whereas odalasvir with/without simeprevir increased ALS‐022227 AUC_0‐24 h_ 1.5‐fold. AL‐335 had no effect on odalasvir or simeprevir pharmacokinetics. Odalasvir and simeprevir AUC_0‐24 h_ increased 1.5‐ to 2‐fold for both drugs when coadministered irrespective of AL‐335 coadministration. Study medications were well tolerated with no serious adverse events. One subject prematurely discontinued study drugs (unrelated event). This study defined the preliminary pharmacokinetic and safety profiles of the combination of AL‐335, odalasvir, and simeprevir in healthy subjects. These data support the further evaluation of this combination for the treatment of chronic hepatitis C virus infection.

**Abbreviations**
AE: adverse event
ALT: alanine aminotransferase
ANOVA: analysis of variance
ANSM: French National Agency for the Safety of Medicine and Health Products
AST: aspartate aminotransferase
AUC_0‐24 h_: area under plasma concentration‐time curve over 24 hours
BCRP: breast cancer resistance protein
BMI: body mass index
CI: confidence interval
*C*_max_: maximum observed plasma concentration
*C*_min_: minimum observed plasma concentration
CPP: Comité de Protection des Personnes
CYP: cytochrome P450
DAA: direct‐acting antiviral
DDI: drug‐drug interaction
ECG: electrocardiogram
HCV: hepatitis C virus
LSM: least squares mean
NA: not applicable
OATP: organic anion‐transporting polypeptide
ODV: odalasvir
PK: pharmacokinetic
QD: once daily
RNA: ribonucleic acid
SMV: simeprevir
SVR12: sustained virological response rates 12 weeks after the end of treatment
*t*_1/2_: apparent elimination half‐life
TEAE: treatment‐emergent adverse event
*T*_last_: time to last measurable plasma concentration
*T*_max_: time of the maximum observed plasma concentration
ULN: upper limit of normal

## 1 INTRODUCTION

According to recently updated World Health Organization estimates, approximately 71 million people worldwide are chronically infected with hepatitis C virus (HCV).^1^ Although 15%‐45% of HCV‐infected patients clear the virus spontaneously without treatment within 6 months of infection, the majority of patients develop chronic HCV infection. This is associated with a spectrum of liver‐related diseases ranging from mild inflammation to liver fibrosis, extrahepatic manifestations, and ultimately cirrhosis. HCV‐related cirrhosis is associated with serious complications including hepatocellular carcinoma.^1^, ^2^ Almost 400 000 patients die each year from complications of chronic HCV infection, with the majority of deaths attributable to cirrhosis and hepatocellular carcinoma.^1^

Current treatment options for HCV infection comprise a combination of two or three oral, direct‐acting antiviral (DAA) compounds.^3^, ^4^ Evidence suggests that adding a third DAA with a different mechanism of action may increase efficacy and allow for a shorter treatment duration.^5^, ^6^, ^7^, ^8^, ^9^, ^10^

In recent studies evaluating 3‐DAA regimens that include a nucleotide analog NS5B inhibitor (eg, sofosbuvir or dasabuvir), sustained virological response rates 12 weeks after the end of treatment (SVR12) were 80%‐98% in treatment‐naïve patients with or without cirrhosis following 6 or 8 weeks of therapy^7^, ^8^, ^9^, ^11^ and 96%‐98% in patients treated for 12‐16 weeks who had previously failed on an NS5A‐containing DAA regimen.^6^, ^10^ An 8‐ and 12‐week 2‐DAA regimen with glecaprevir (NS3/4A protease inhibitor) and pibrentasvir (NS5A inhibitor) has also been evaluated in treatment‐naïve or ‐experienced noncirrhotic genotype 1‐infected patients demonstrating 99% and 100% SVR12 rates, respectively.^12^

It has been suggested that the high SVR12 rates achieved with nucleotide‐based 3‐DAA regimens following a shortened treatment duration of 6 or 8 weeks may be partly ascribed to the more rapid first‐phase decline in HCV ribonucleic acid (RNA) observed when a third DAA was added.^8^ In addition to the high levels of therapeutic efficacy achieved with 2‐ or 3‐DAA regimens, the opportunity to provide patients with an effective shorter course of therapy could also have a favorable impact on affordability, adherence, and patient quality of life due to reduced side effects and decreased pill burden.^8^, ^13^, ^14^

Simeprevir is a HCV NS3/4A protease inhibitor licensed for the treatment of chronic HCV infection as a component of combination antiviral therapy. The recommended adult dose of simeprevir is 150 mg (100 mg in Japan) once daily (QD) administered with food. Simeprevir is a substrate and inhibitor of P‐glycoprotein, breast cancer resistance protein (BCRP), and organic anion‐transporting polypeptide (OATP) 1B1/3, and is transported into the liver by OATP1B1/3 where it undergoes metabolism by cytochrome P450 (CYP) 3A. It is also a mild inhibitor of intestinal (not hepatic) CYP3A and a mild but not clinically relevant inhibitor of CYP1A2.^15^ Coadministration of simeprevir with cyclosporine, an inhibitor of multiple transporters such as OATP and P‐glycoprotein, and with CYP3A inhibitors and inducers such as ritonavir and efavirenz, has resulted in clinically significant drug interactions such that coadministration of simeprevir with these drugs is not recommended.^15^

Odalasvir is an investigational HCV NS5A inhibitor in development for the treatment of HCV infection. It is primarily cleared by biliary secretion and undergoes minimal metabolism. In single‐dose pharmacokinetic studies, the terminal elimination half‐life was approximately 250 hours. No clinically significant drug‐drug interactions (DDIs) were observed between odalasvir and montelukast (CYP2C8 substrate), atazanavir/ritonavir, efavirenz/emtricitabine/tenofovir disoproxil fumarate, darunavir/ritonavir, or raltegravir (UGT1A1 substrate). Odalasvir is a substrate of OATP1B1 and a P‐glycoprotein inhibitor in vivo and in vitro (data on file).

AL‐335 is an investigational uridine‐based NS5B inhibitor in development for the treatment of HCV infection. AL‐335 is rapidly converted to ALS‐022399 (monophosphate precursor) by esterases then subsequently phosphorylated intracellularly. The active moiety of AL‐335, ALS‐022235 (5′‐triphosphate), inhibits HCV NS5B RNA‐dependent polymerase by acting as a chain terminator of RNA synthesis. AL‐335 and its metabolites do not interact with host polymerases, including the mitochondrial RNA polymerase. Dephosphorylation of ALS‐022235 yields the parent nucleoside ALS‐022227.^16^ Both AL‐335 and its metabolites have low inhibition potential for CYPs 1A2, 2B6, 2C8, 2C9, 2C19, 2D6, and 3A4, and are therefore not expected to interact with drugs that are metabolized by the CYP enzymes. AL‐335 is a substrate of BCRP and P‐glycoprotein, but does not inhibit P‐glycoprotein; it is neither a substrate nor an inhibitor of OATP1B1/1B3. Furthermore, the metabolites ALS‐022399 and ALS‐022227 are neither substrates nor inhibitors of P‐glycoprotein, or OATP1B1/1B3 (data on file).

With their different mechanisms of action, combining a nucleotide analog (e.g. AL‐335) with an NS5A inhibitor (eg, odalasvir) with or without an NS3A/4 protease inhibitor (eg, simeprevir) may be a potentially effective combination that allows for short duration treatment (eg, ≤8 weeks) of chronic HCV infection. However, administering three DAAs in combination may give rise to the development of complex DDIs that can affect the concentrations of coadministered drugs.^17^, ^18^ Determining the potential for DDIs is therefore important to inform decisions regarding dose selection of each of the components within a given combination.

The current Phase I study (ClinicalTrials.gov ID: NCT02512562; Eudra CT: 2015‐002074‐20) was conducted to investigate the effect of multiple oral doses of odalasvir and simeprevir, separately and in combination, on the pharmacokinetics of AL‐335 and its metabolites (ALS‐022399 and ALS‐022227) when given as multiple oral doses to healthy subjects to guide dose selection of the individual components for the development of a 2‐ or 3‐DAA combination regimen for the treatment of HCV infection. The effect of odalasvir and simeprevir on the pharmacokinetics of each other, and the effect of AL‐335 and its metabolites on the pharmacokinetics of odalasvir and simeprevir were also evaluated. In addition, the safety and tolerability of the 2‐ and 3‐DAA combination regimen was also assessed.

## 2 MATERIAL AND METHODS

### 2.1 Study design and subjects

This was an open‐label, two‐group, fixed‐sequence study conducted at a single study center in Rennes, France (Biotrial). A two‐group, fixed‐sequence study design was chosen as this was deemed to be the most efficient way to evaluate the effect of simeprevir with/without odalasvir on AL‐335; and vice versa (Group 1) and the effect of odalasvir with/without simeprevir on AL‐335; and vice versa (Group 2). Thus, this study design had the potential to gather both 2‐way and 3‐way DDIs. For both groups, the study consisted of a screening period, a study period, and two follow‐up visits. The study was authorized by the French National Agency for the Safety of Medicine and Health Products (ANSM) and was approved by an Independent Ethics Committee (Comité de Protection des Personnes [CPP] Ouest VI, Brest, France; Reference CPP Ouest 6–CPP 888/MS1), and was conducted in accordance with the principles of the Declaration of Helsinki and International Conference on Harmonization. All subjects provided written informed consent to participate. Drug and molecular target nomenclature in this manuscript conforms to the *British Journal of Pharmacology*'s Concise Guide to Pharmacology 2017/18.^19^

No formal sample size calculations were performed. Enrolment of approximately 32 healthy subjects was planned, providing 16 subjects in each group to ensure at least 12 evaluable subjects per group. Eligible subjects were healthy subjects, as indicated by the absence of chronic viral infections (absence of serological evidence of active infection with hepatitis B or C virus or human immunodeficiency virus) or other chronic conditions, aged 18‐60 years with a body mass index of 18‐32 kg/m^2^. Full inclusion/exclusion criteria are provided in the Supplementary Appendix.

Concomitant use of prescription and over‐the‐counter medications including vitamins, herbal medications, dietary supplements, sedatives, hypnotics, and antihistamines was not permitted, with the exception of paracetamol (acetaminophen) which was permitted on an occasional basis. Consumption of grapefruit (juice), Seville orange (juice), and other inhibitors/inducers of CYP450 enzyme and P‐glycoprotein drug transporters, substrates of CYP3A and P‐glycoprotein was not permitted within 14 days prior to administration of study drugs and throughout the study until follow‐up. Consumption of alcohol was prohibited and caffeine intake was limited to ≤2 cups of caffeine‐containing beverage per day. History of tobacco use or using nicotine‐containing products within 3 months of the screening visit was prohibited.

**Figure 1:**
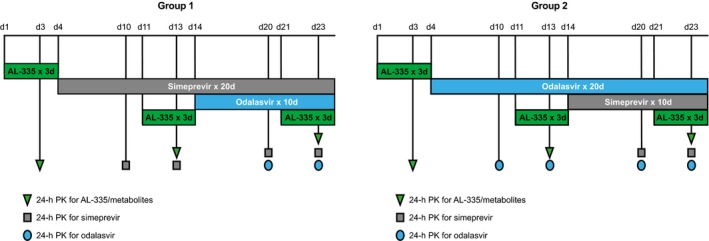
Study design. *PK* pharmacokinetics

The study design is shown in Figure 1. Subjects in Group 1 received simeprevir 150 mg QD on days 4‐23, odalasvir 150 mg (loading dose) on day 14, odalasvir 50 mg QD on days 15‐23, and AL‐335 800 mg QD on days 1‐3, 11‐13, and 21‐23. Subjects in Group 2 received odalasvir 150 mg (loading dose) on day 4, odalasvir 50 mg QD on days 5‐23, simeprevir 150 mg QD on days 14‐23, and AL‐335 800 mg QD on days 1‐3, 11‐13, and 21‐23. The doses chosen for evaluation in this study were either the highest approved dose (simeprevir) or doses found to have substantial antiviral effects in prior studies (AL‐335 and odalasvir).^16^, ^20^ For odalasvir, a loading dose was utilized to shorten the time to reach steady‐state. A minimum of 10 days was considered sufficient for both odalasvir and simeprevir to reach steady‐state. Due to the shorter half‐life of AL‐335 and its metabolites, 3 days was considered sufficient to achieve steady‐state.

All study treatments were administered on‐site orally under fed conditions (a standard diet comprising 55% carbohydrates, 30% fat, and 15% protein; ~2470 kcal on days where pharmacokinetic samples were taken). A visual check of the mouth was done to confirm study treatments were taken and swallowed. Subjects were assigned sequentially to Group 1 then to Group 2 after confirming eligibility.

The primary objective of the study was to assess the 2‐ and 3‐way interaction between AL‐335, odalasvir, and simeprevir in healthy subjects. A secondary objective was to determine the safety and tolerability of the DAAs alone and in combination.

### 2.2 Study assessments

In both Groups 1 and 2, subjects were admitted to the clinic on day ‐1. Subjects remained in the clinic for the duration of the study with the exception of days 5‐8 and days 15‐18, where daily outpatient visits were performed. Subjects were discharged on day 24. The last study assessments were performed at the final follow‐up visit 28 days after completion of the study confinement period.

#### 2.2.1 Bioanalysis and pharmacokinetic analysis

As this was an observational pharmacokinetic study, blood samples for pharmacokinetic analysis were collected predose and at 0.25, 0.5, 1, 2, 3, 4, 6, 8, 10, 12, and 24 hours postdose on days 3, 13, and 23 for determination of AL‐335, ALS‐022399, and ALS‐022227 plasma concentrations (Groups 1 and 2). For simeprevir, blood samples for determination of simeprevir plasma concentrations were taken at the same time points but on days 10, 13, 20, and 23 (Group 1) or days 20 and 23 (Group 2). Blood samples for the determination of odalasvir plasma concentrations were collected predose and at 1, 3, 4, 5, 6, 7, 8, 9, 10, 12, 16, and 24 hours postdose on days 20 and 23 (Group 1) or days 10, 13, 20, and 23 (Group 2), with two further samples also collected on the day 7 and day 28 follow‐up assessments (both groups).

Plasma concentrations of AL‐335, ALS‐022399, ALS‐022227, odalasvir, and simeprevir were determined using validated liquid chromatography‐tandem mass spectrometry methods. Dichlorvos solution was added to blood samples from AL‐335‐treated subjects to stabilize AL‐335 and its metabolites (25 μL of 5 mmol/L dichlorvos into 1 mL blood). The lower limit of quantification was 1 ng/mL, 2 ng/mL, and 5 ng/mL for AL‐335, ALS‐022399, and ALS‐022227, respectively, 1 ng/mL for odalasvir, and 2 ng/mL for simeprevir.

Pharmacokinetic analyses were performed on all subjects with available profiles who received at least one dose of study drug without any protocol deviation affecting pharmacokinetic evaluation. Pharmacokinetic parameters of interest included maximum observed plasma concentration (*C*_max_) and area under plasma concentration‐time curve over 24 hours (AUC_0‐24 h_). Secondary parameters were last observed plasma concentration, apparent elimination half‐life, time of the maximum observed plasma concentration (*T*_max_), and time to last measurable plasma concentration (*T*_last_). Pharmacokinetic parameters for AL‐335, ALS‐022399, ALS‐022227, simeprevir, and odalasvir were calculated using standard noncompartmental methods. AUCs were calculated using linear‐linear trapezoidal summation. Analysis of variance (ANOVA) was applied to the log‐transformed parameters and evaluated using a mixed‐effects model, including treatment as fixed effect and subject as random effect. Least squares mean ratios of *C*_max_, AUC_0‐24 h,_ and minimum observed plasma concentration (*C*_min_), and corresponding 90% confidence intervals (CI) were constructed, comparing the test *vs*. reference treatments. The equivalence between each treatment group was concluded if the 90% CI was within 80.00‐125.00%. The Wilcoxon signed‐rank test was used to assess *T*_max_ and a one‐way ANOVA was used to assess steady‐state on factor ‘day’. Pharmacokinetic data were analyzed using Phoenix^®^ WinNonlin^®^ version 6.4 (Certara, Princeton, NJ, USA) and SAS^®^ software version 9.3 (SAS Institute, Inc., Cary, NC, USA).

#### 2.2.2 Safety and tolerability analysis

Safety and tolerability were evaluated throughout the study until the last follow‐up visit. Blood samples for serum chemistry, hematology, and urinalysis were collected at screening, days −1, 2, 4, 10, 12, 13, 19, 21, 23, and 24 and days 7 and 28 of follow‐up. Complete physical examination was conducted at screening, day 1, and day 24, and days 7 and 28 of follow‐up. Vital‐sign assessments were performed at screening, days −1, 1‐4, 9‐14, and 19‐24, and days 7 and 28 of follow‐up. Electrocardiograms (ECGs) were performed at screening and day −1, and then daily from days 1‐24 and at days 7 and 28 of follow‐up visits. All adverse events (AEs) and serious AEs were coded using the Medical Dictionary for Regulatory Activities (version 18.0).

Safety analyses were performed on all subjects who received at least one dose of study drug, including those who did not complete the study.

Descriptive statistics of the safety findings were made using SAS^®^ software version 9.3 (SAS Institute, Inc., Cary, NC, USA).

Unless otherwise stated, statistical tests for analyses were two‐sided at a 5% level of significance.

## 3 RESULTS

### 3.1 Subjects

**Figure 2:**
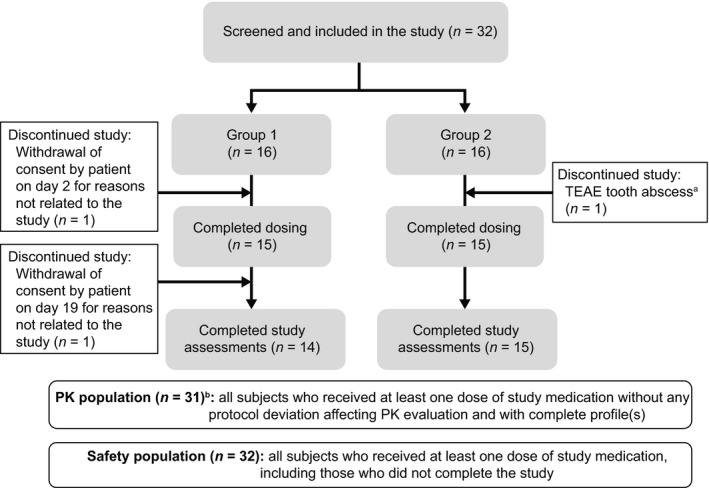
Subject disposition. *PK* pharmacokinetic; *QD* once daily; *TEAE* treatment‐emergent adverse event. ^a^Risk of interaction between treatment for tooth abscess and study drugs. ^b^The subject that withdrew from the study on day 2, for reasons not related to the study, received two QD administrations of AL‐335 800 mg alone (day 1 and day 2) before any PK samples were taken and was therefore excluded from the PK analysis

A total of 54 subjects were screened; 22 subjects were not included due to screening failures. Thirty‐two healthy subjects were screened and included in the study; of these, 29 (91%) completed the study and 3 (9%) discontinued prematurely (Figure 2). Two subjects in Group 1 discontinued due to reasons not related to the study; one withdrew from the study on day 2 and thus received two QD administrations of AL‐335 800 mg alone (day 1 and day 2) before any pharmacokinetic samples were taken and was therefore excluded from the pharmacokinetic analysis; the other subject withdrew from the study on day 19 and was included in the pharmacokinetic analysis. The third subject from Group 2 discontinued treatment early on day 11 due to a tooth abscess and was included in the pharmacokinetic analysis.

All 32 subjects were included in the safety analysis population and 31 were included in the pharmacokinetic analysis.

Subject demographic and baseline disease characteristics are summarized in Table 1. All 32 enrolled subjects (100%) were male, the majority were white (94%), mean age (±SD) was 39.0 ± 13.3 years, and mean body mass index (±SD) was 24.8 ± 3.8 kg/m^2^.

**Table 1:** Subject baseline characteristics

	Group 1 (N = 16)	Group 2 (N = 16)	Overall (N = 32)
Mean age, years (±SD)	36.1 (13.7)	41.9 (12.6)	39.0 (13.3)
Male, n (%)	16 (100)	16 (100)	32 (100)
Race, n (%)			
White, n (%)	15 (93.8)	15 (93.8)	30 (93.8)
Black or African American, n (%)	1 (6.3)	0 (0.0)	1 (3.1)
Mixed race, n (%)	0 (0.0)	1 (6.3)	1 (3.1)
Ethnicity			
Not Hispanic or Latino, n (%)	15 (93.8)	16 (100)	31 (96.9)
Hispanic or Latino, n (%)	1 (6.3)	0 (0.0)	1 (3.1)
Mean weight, kg (±SD)	75.6 (12.8)	78.6 (14.7)	77.1 (13.7)
Mean BMI, kg/m^2^ (±SD)	23.6 (3.6)	25.9 (3.8)	24.8 (3.8)
Current or former smoker, n (%)^a^	9 (56.3)	3 (18.8)	12 (37.5)
Currently or formerly consumed alcohol, n (%)^a^	3 (18.8)	3 (18.8)	6 (18.8)

BMI, body mass index; SD, standard deviation.

a No subject smoked, consumed alcohol, or consumed grapefruit or Seville orange during the study.

### 3.2 Pharmacokinetics

Odalasvir and simeprevir, individually or in combination, had a marked effect on the pharmacokinetic profile of AL‐335 and its metabolite ALS‐022399, but less of an effect on ALS‐022227 (Tables 2, 3, 4).

**Table 2:** Summary of pharmacokinetic parameters for AL‐335^a^

	Group 1^b^			Group 2^b^		
	Day 3	Day 13	Day 23	Day 3	Day 13	Day 23
	AL‐335 alone	AL‐335 + SMV	AL‐335 + ODV + SMV	AL‐335 alone	AL‐335 + ODV	AL‐335 + ODV + SMV
*C*_max_ (ng/mL)	162 (77)	376 (186)	747 (340)	151 (72)	516 (259)	950 (472)
N	15	15	14	16	15	15
LSM ratio^c^	–	2.29 (1.79‐2.93)	4.60 (3.68‐5.75)	–	3.41 (2.51‐4.64)	6.30 (4.93‐8.05)
*T*_max_ (h)	2.00 (0.50‐3.00)	2.00 (0.50‐4.00)	2.00 (1.00‐4.00)	2.00 (0.50‐4.00)	2.00 (0.50‐4.00)	3.00 (1.00‐4.00)
N	15	15	14	16	15	15
C_last_ (ng/mL)	6 (8)	3 (2)	3 (1)	3 (2)	2 (1)	2 (1)
N	15	15	14	16	15	15
*T*_last_ (h)	6.00 (4.00‐6.00)	8.00 (6.00‐10.00)	8.00 (6.00‐10.08)	6.00 (4.00‐8.00)	8.00 (4.00‐12.00)	10.00 (8.00‐12.03)
N	15	15	14	16	15	15
AUC_0‐24 h_ (ng.h/mL)	295 (11)	1,002 (520)	2,084 (1,063)	324 (130)	1,332 (604)	2,919 (1,670)
N	9	12	14	12	14	15
LSM ratio^c^	–	2.92 (2.26‐3.77)	6.92 (5.46‐8.77)	–	3.97 (2.96‐5.33)	8.23 (6.26‐10.82)
*t*_1/2_ (h)	0.61 (0.09)	0.64 (0.09)	0.69 (0.10)	0.69 (0.17)	0.62 (0.08)	0.84 (0.11)
N	9	12	14	12	14	15

AUC_0‐24 h,_ area under the plasma concentration‐time curve over 24 h; CI: confidence interval; *C*_last_, last measurable plasma concentration; *C*_max_, maximum observed plasma concentration; LSM, least squares mean; ODV, odalasvir; QD, once daily; SMV, simeprevir; *t*_1/2_ apparent elimination half‐life; *T*_last_, time to last measurable plasma concentration; *T*_max_, time of maximum observed plasma concentration.

a Unless otherwise stated, data for *C*_max_, *C*_last_, AUC_0‐24 h_, and t_1/2_ are shown as arithmetic mean ± standard deviation and data for *T*_max_ and *T*_last_ are median (range). No analysis was performed on *C*_min_ as all values were equal to 0.

b Group 1 received SMV 150 mg QD on days 4‐23, ODV 150 mg (loading dose) on day 14, ODV 50 mg QD on days 15‐23, and AL‐335 800 mg QD on days 1‐3, 11‐13, and 21‐23. Group 2 received ODV 150 mg (loading dose) on day 4, ODV 50 mg QD on days 5‐23, SMV 150 mg QD on days 14‐23, and AL‐335 800 mg QD on days 1‐3, 11‐13, and 21‐23.

c Point estimate for LSM ratio (90% CI) for AL‐335 + ODV or AL‐335 + SMV + ODV versus AL‐335 alone.

**Table 3:** Summary of pharmacokinetic parameters for ALS‐022399^a^

	Group 1^b^			Group 2^b^		
	Day 3	Day 13	Day 23	Day 3	Day 13	Day 23
	AL‐335 alone	AL‐335 + SMV	AL‐335 + ODV + SMV	AL‐335 alone	AL‐335 + ODV	AL‐335 + ODV + SMV
*C*_max_ (ng/mL)	57 (14)	103 (35)	163 (46)	58 (24)	130 (44)	194 (73)
N	15	15	14	16	15	15
LSM ratio^c^	‐	1.74 (1.48‐2.05)	2.79 (2.50‐3.13)	–	2.31 (1.94‐2.75)	3.37 (2.86‐3.97)
*T*_max_ (h)	3.00 (2.00‐6.00)	3.00 (2.00‐4.00)	3.00 (2.00‐6.00)	3.50 (1.00‐4.03)	3.00 (1.00‐4.00)	3.00 (2.00‐4.00)
N	15	15	14	16	15	15
*C*_last_ (ng/mL)	6 (4)	8 (4)	10 (4)	6 (4)	9 (4)	9 (4)
N	15	15	14	16	15	15
*T*_last_ (h)	12.00 (12.00‐12.00)	12.00 (12.00‐12.02)	12.00 (12.00‐12.17)	12.00 (12.00‐23.97)	12.00 (12.00‐12.05)	12.00 (12.00‐24.00)
N	15	15	14	16	15	15
AUC_0‐24 h_ (ng.h/mL)	324 (70)	541 (155)	852 (226)	336 (92)	637 (159)	953 (343)
N	13	14	13	14	14	15
LSM ratio^c^	–	1.62 (1.42‐1.85)	2.56 (2.27‐2.89)	–	1.92 (1.66‐2.21)	2.78 (2.42‐3.21)
*t*_1/2_ (h)	2.66 (0.70)	2.35 (0.62)	2.08 (0.32)	3.23 (2.18)	2.17 (0.50)	2.06 (0.42)
N	13	14	13	14	14	15

AUC_0‐24 h,_ area under the plasma concentration‐time curve over 24 h; CI, confidence interval; *C*_last,_ last measurable plasma concentration; *C*_max_, maximum observed plasma concentration; LSM*,* least squares mean; ODV, odalasvir; SMV, simeprevir; *t*_1/2_, apparent elimination half‐life; *T*_last_, time to last measurable plasma concentration; *T*_max_, time of maximum observed plasma concentration.

a Unless otherwise stated, data for *C*_max_, *C*_last_, AUC_0‐24 h_, and *t*_1/2_ are shown as arithmetic mean ± SD and data for *T*_max_ and *T*_last_ are median (range). No analysis was performed on *C*_min_ as all values were equal to 0.

b Group 1 received SMV 150 mg QD on days 4‐23, ODV 150 mg (loading dose) on day 14, ODV 50 mg QD on days 15‐23, and AL‐335 800 mg QD on days 1‐3, 11‐13, and 21‐23. Group 2 received ODV 150 mg (loading dose) on day 4, ODV 50 mg QD on days 5‐23, SMV 150 mg QD on days 14‐23, and AL‐335 800 mg QD on days 1‐3, 11‐13, and 21‐23.

c Point estimate for LSM ratio (90% CI) for AL‐335 + ODV or AL‐335 + SMV + ODV versus AL‐335 alone.

**Table 4:** Summary of pharmacokinetic parameters for ALS‐022227^a^

	Group 1^b^			Group 2^b^		
	Day 3	Day 13	Day 23	Day 3	Day 13	Day 23
	AL‐335 alone	AL‐335 + SMV	AL‐335 + ODV + SMV	AL‐335 alone	AL‐335 + ODV	AL‐335 + ODV + SMV
C_max_ (ng/mL)	614 (234)	524 (145)	500 (186)	416 (160)	471 (147)	448 (146)
N	15	15	14	16	15	15
LSM ratio^c^	–	0.88 (0.77‐1.00)	0.81 (0.69‐0.95)	–	1.16 (0.99‐1.36)	1.10 (0.93‐1.29)
T_max_ (h)	4.00 (3.00–4.02)	4.00 (2.00‐6.00)	4.00 (3.00‐6.00)	4.00 (3.00‐6.00)	4.00 (3.00‐6.00)	4.00 (3.00‐6.02)
N	15	15	14	16	15	15
C_min_ (ng/mL)	16 (3)	29 (8)	52 (16)	0 (0)	0 (0)	0 (0)
N	15	15	14	16	15	15
LSM ratio^c^	–	1.76 (1.58, 1.96)	3.03 (2.58, 3.56)	–	2.68 (2.38, 3.02)	3.51 (3.06, 4.01)
AUC_0‐24 h_ (ng.h/mL)	3551 (940)	3617 (881)	4029 (1,101)	2826 (870)	4187 (1220)	4325 (1347)
N	15	15	14	16	15	15
LSM ratio^c^	–	1.02 (0.91‐1.15)	1.13 (0.99‐1.29)	–	1.49 (1.32‐1.68)	1.54 (1.35‐1.74)
*t*_1/2_ (h)^d^	6.0 (0.5)	NA	NA	NA	NA	NA
N	8	NA	NA	NA	NA	NA

AUC_0‐24 h_, area under the plasma concentration‐time curve over 24 h; CI, confidence interval; *C*_max_, maximum observed plasma concentration; *C*_min_, minimum observed plasma concentration; LSM, least squares mean; NA, not applicable; ODV, odalasvir; SMV, simeprevir; *t*_1/2_, apparent elimination half‐life; *T*_max_, time of maximum observed plasma concentration.

a Unless otherwise stated, data for *C*_max_, *C*_min,_ *C*_last_, AUC_0‐24 h_, and *t*_1/2_ are shown as arithmetic mean ± SD and data for *T*_max_ and *T*_last_ are median (range).

b Group 1 received SMV 150 mg QD on days 4‐23, ODV 150 mg (loading dose) on day 14, ODV 50 mg QD on days 15‐23, and AL‐335 800 mg QD on days 1‐3, 11‐13, and 21‐23. Group 2 received ODV 150 mg (loading dose) on day 4, ODV 50 mg QD on days 5‐23, SMV 150 mg QD on days 14‐23, and AL‐335 800 mg QD on days 1‐3, 11‐13, and 21‐23.

c Point estimate for LSM ratio (90% CI) for AL‐335 + ODV or AL‐335 + SMV + ODV versus AL‐335 alone.

d The mean *t*_1/2_ could not be reliably determined (over a sufficient time interval) for all treatment groups, except in Group 1 following administration of AL‐335 alone.

**Figure 3:**
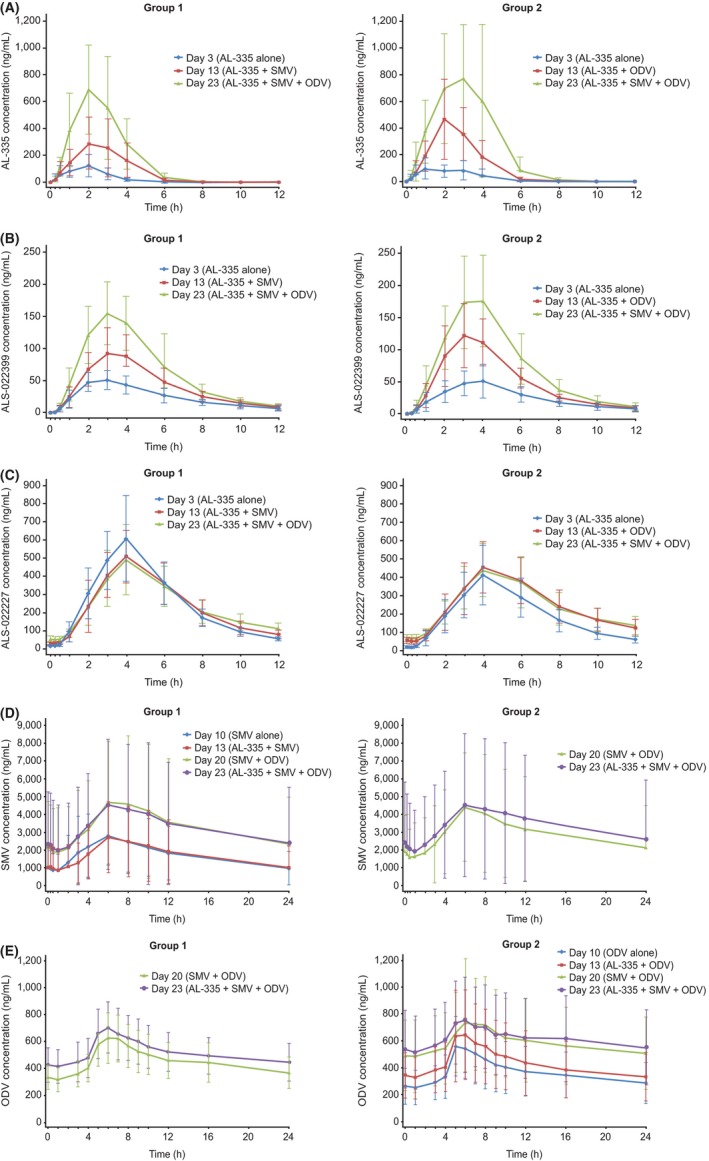
Mean (±SD ) plasma concentration‐time curve of (A) AL‐335; (B) ALS‐022399; (C) ALS‐022227; (D) simeprevir; and (E) odalasvir by group^a^. ODV, odalasvir; QD*,* once daily; SMV*,* simeprevir. ^a^Group 1 received SMV 150 mg QD on days 4‐23, ODV 150 mg (loading dose) on day 14, ODV 50 mg QD on days 15‐23, and AL‐335 800 mg QD on days 1‐3, 11‐13, and 21‐23. Group 2 received ODV 150 mg (loading dose) on day 4, ODV 50 mg QD on days 5‐23, SMV 150 mg QD on days 14‐23, and AL‐335 800 mg QD on days 1‐3, 11‐13, and 21‐23

A 2.3‐ and 2.9‐fold increase in AL‐335 *C*_max_ and AUC_0‐24 h_, respectively, was observed when AL‐335 was coadministered with simeprevir 150 mg QD (Group 1), and a 3.4‐ and 4.0‐fold increase in AL‐335 *C*_max_ and AUC_0‐24 h_, respectively, was observed when AL‐335 was coadministered with odalasvir 50 mg QD (Group 2). When odalasvir and simeprevir were coadministered together, AL‐335 plasma concentrations increased further: C_max_, 4.6‐6.3‐fold and AUC_0−24 h_, 6.9‐8.2‐fold versus AL‐335 administered alone (Table 2, Figure 3). No clinically relevant difference in AL‐335 *T*_max_ was observed between any of the treatment combinations.

The effect of coadministration with odalasvir and/or simeprevir on ALS‐022399 pharmacokinetics was less marked relative to AL‐335. A 1.6‐ to 2.3‐fold increase in ALS‐022399 *C*_max_ and AUC_0‐24 h_ was observed when AL‐335 was coadministered with odalasvir or simeprevir, and a 2.6‐ to 3.4‐fold increase was noted when AL‐335 was co administered with both odalasvir and simeprevir (Table 3, Figure 3). No statistically significant difference in ALS‐022399 *T*_max_ was observed between any of the treatment combinations.

Simeprevir had no effect on ALS‐022227 *C*_max_ and AUC_0__‐__24 h_ but increased *C*_min_ 1.8‐fold (Table 4, Figure 3). Odalasvir also had no significant effect on ALS‐022227 *C*_max_ but increased AUC_0‐24 h_ and *C*_min_ by 1.5‐ and 2.7‐fold, respectively. The effect of coadministration of both odalasvir and simeprevir on ALS‐022227 *C*_max_ and AUC_0‐24 h_ was variable between Groups 1 and 2 (Group 1: *C*_max_ 19% decrease and no significant effect on AUC_0‐24 h_; Group 2: no significant effect on *C*_max_ and 1.5‐fold increase in AUC_0‐24 h_). However, across both groups, coadministration of odalasvir and simeprevir increased ALS‐022227 C_min_ by 3.0‐ to 3.5‐fold. No clinically relevant difference in *T*_max_ was observed between any of the treatments.

Administration of AL‐335 did not alter the pharmacokinetics of simeprevir or odalasvir. The 90% CIs for the treatment ratios for *C*_max_ and AUC_0‐24 h_ were almost within the bioequivalence range (Tables 5, 6, Figure 3). Following coadministration of odalasvir with or without AL‐335, simeprevir *C*_max_, AUC_0‐24 h_, and *C*_min_ were increased by 1.4‐ to 1.7‐fold, 1.6‐ to 1.8‐fold, and 1.4‐ to 1.8‐fold, respectively (Table 5). No clinically relevant difference in simeprevir *T*_max_ was observed when simeprevir was coadministered with odalasvir. Coadministration of simeprevir with or without AL‐335 increased odalasvir *C*_max_, AUC_0‐24 h_, and *C*_min_ by 1.3‐fold, 1.5‐ to 1.6‐fold, and 1.9‐ to 2.1‐fold, respectively (Table 6). In Group 2, a statistically significant difference in odalasvir *T*_max_ was observed between simeprevir coadministered with odalasvir (median *T*_max_ 7.00; range 3.0‐9.0) versus odalasvir alone (median *T*_max_ 5.00; range: 5.0‐7.0) (*P *=* *.0116) and with coadministration of AL‐335, odalasvir, and simeprevir (median *T*_max_ 6.00; range: 5.0‐16.0) versus odalasvir alone (median *T*_max_ 5.00; range: 5.0‐7.0) (*P *=* *.0176); no statistically significant difference in odalasvir *T*_max_ was observed between AL‐335 coadministered with odalasvir versus odalasvir alone.

**Table 5:** Summary of pharmacokinetic parameters for simeprevir^a^

	Group 1^b^				Group 2^b^	
	Day 10	Day 13	Day 20	Day 23	Day 20	Day 23
	SMV alone	SMV + AL‐335	SMV + ODV	SMV + AL‐335 + ODV	SMV + ODV	SMV + AL‐335 + ODV
C_max_ (ng/mL)	2957 (1850)	2764 (2000)	5219 (3651)	4741 (3838)	4529 (3290)	4951 (4089)
N	15	15	14	14	15	15
LSM ratio^c^	–	0.89 (0.79‐1.00)	1.66 (1.36‐2.01)	1.43 (1.23‐1.66)	–	–
*T*_max_ (h)	6.00 (3.0‐8.0)	6.00 (3.0‐10.0)	6.00 (4.1‐23.9)	6.00 (4.0‐10.0)	6.00 (4.0‐8.0)	8.00 (4.0‐12.0)
N	15	15	14	14	15	15
*C*_min_ (ng/mL)	833 (856)	857 (1129)	1446 (2064)	1910 (2441)	1274 (1035)	1847 (2313)
N	15	15	14	14	15	15
LSM ratio^c^	–	0.88 (0.76‐1.02)	1.41 (1.01‐1.99)	1.82 (1.52‐2.19)	–	–
*C*_last_ (ng/mL)	981 (935)	1015 (1199)	2337 (2626)	2391 (3127)	2139 (2348)	2593 (3333)
N	15	15	14	14	15	15
*T*_last_ (h)	23.9 (23.9‐24.0)	23.9 (23.9‐24.0)	23.9 (23.9‐24.0)	24.0 (24.0‐24.0)	23.9 (23.8‐24.1)	24.0 (24.0‐24.1)
N	15	15	14	14	15	15
AUC_0‐24 h_ (ng.h/mL)	41 157 (32 823)	40 528 (37 247)	78 022 (70 261)	77 829 (78 399)	69 901 (62 925)	81 030 (81 138)
N	15	15	14	14	15	15
LSM ratio^c^	–	0.93 (0.83‐1.05)	1.76 (1.53‐2.01)	1.60 (1.38‐1.85)	–	–

AUC_0‐24* *h_, area under the plasma concentration‐time curve over 24 h; CI, confidence interval; *C*_last_, last measurable plasma concentration; *C*_max_, maximum observed plasma concentration; *C*_min_, minimum observed plasma concentration; LSM, least squares mean; ODV, odalasvir; SMV, simeprevir; *t*_1/2_, apparent elimination half‐life; *T*_last,_ time to last measurable plasma concentration; *T*_max_, time of maximum observed plasma concentration.

a Unless otherwise stated, data for *C*_max_, *C*_min,_ *C*_last_, and AUC_0‐24 h_, are shown as arithmetic mean ± standard deviation and data for *T*_max_ and *T*_last_ are median (range).

b Group 1 received SMV 150 mg QD on days 4‐23, ODV 150 mg (loading dose) on day 14, ODV 50 mg QD on days 15‐23, and AL‐335 800 mg QD on days 1‐3, 11‐13, and 21‐23. Group 2 received ODV 150 mg (loading dose) on day 4, ODV 50 mg QD on days 5‐23, SMV 150 mg QD on days 14‐23, and AL‐335 800 mg QD on days 1‐3, 11‐13, and 21‐23.

c Point estimate for LSM ratio (90% CI) for SMV + AL‐335, SMV + ODV or SMV + AL‐335 + ODV versus SMV alone.

**Table 6:** Summary of pharmacokinetic parameters for odalasvir^a^

	Group 1^b^		Group 2^b^			
	Day 20	Day 23	Day 10	Day 13	Day 20	Day 23
	ODV + SMV	ODV + SMV + AL‐335	ODV alone	ODV + AL‐335	ODV + SMV	ODV + SMV + AL‐335
*C*_max_ (ng/mL)	649 (183)	717 (192)	582 (221)	669 (349)	770 (346)	780 (321)
N	14	14	16	15	15	15
LSM ratio^c^	–	–	–	1.06 (0.95‐1.17)	1.25 (1.11‐1.41)	1.28 (1.17‐1.40)
*T*_max_ (h)	6.51 (5.0‐8.0)	6.00 (5.0‐8.0)	5.00 (5.0‐7.0)	5.05 (5.0‐8.0)	7.00 (3.0‐9.0)	6.00 (5.0‐16.0)
N	14	14	16	15	15	15
*C*_min_ (ng/mL)	313 (89)	398 (116)	235 (132)	314 (166)	471 (263)	508 (270)
N	14	14	16	15	15	15
LSM ratio^c^	–	–	–	1.30 (1.11‐1.52)	1.91 (1.63‐2.24)	2.09 (1.78‐2.45)
C_last_ (ng/mL)	368 (116)	55 (21)	287 (150)	331 (180)	507 (268)	90 (53)
N	14	14	16	15	15	15
*T*_last_ (h)	23.9 (23.9‐24.0)	696.0 (696.0‐696.0)	23.9 (23.9‐24.0)	23.9 (23.9‐23.9)	23.9 (23.8‐24.1)	696.0 (696.0‐815.0)
N	14	14	16	15	15	15
AUC_0‐24 h_ (ng.h/mL)	10 734 (3124)	12 376 (3407)	8694 (3956)	10 210 (5252)	13 908 (6911)	14 771 (7090)
N	14	14	16	15	15	15
LSM ratio^c^	–	–	–	1.10 (1.01‐1.20)	1.50 (1.36‐1.67)	1.61 (1.46‐1.77)
*t*_1/2_ (h)^c^	NA	213.13 (15.9)	NA	NA	NA	233.9 (35.5)
N	NA	11	NA	NA	NA	9

AUC_0‐24 h,_ area under the plasma concentration‐time curve over 24 h; CI*,* confidence interval; *C*_last_, last measurable plasma concentration; *C*_max_, maximum observed plasma concentration; *C*_min_, minimum observed plasma concentration; LSM*,* least squares mean; NA, not applicable; ODV, odalasvir; SMV, simeprevir; *t*_1/2*,*_ apparent elimination half‐life; *T*_last_, time to last measurable plasma concentration; *T*_max_, time of maximum observed plasma concentration.

a Unless otherwise stated, data for *C*_max_, *C*_min,_ *C*_last_, AUC_0‐24 h_, and *t*_1/2_ are shown as arithmetic mean ± SD and data for *T*_max_ and *T*_last_ are median (range).

b Group 1 received SMV 150 mg QD on days 4‐23, ODV 150 mg (loading dose) on day 14, ODV 50 mg QD on days 15‐23, and AL‐335 800 mg QD on days 1‐3, 11‐13, and 21‐23. Group 2 received ODV 150 mg (loading dose) on day 4, ODV 50 mg QD on days 5‐23, SMV 150 mg QD on days 14‐23, and AL‐335 800 mg QD on days 1‐3, 11‐13, and 21‐23.

Point estimate for LSM ratio (90% CI) for ODV + AL‐335, ODV + SMV or ODV + AL‐335 + SMV versus ODV alone.

c Evaluation of the mean *t*_1/2_ was performed on day 23 only, when enough detectable time points during the elimination phase were available.

### 3.3 Safety

A total of 20 treatment‐emergent AEs (TEAEs) were reported in 12 subjects (37.5%) (Table 7). None of these TEAEs were considered serious. One TEAE (tooth abscess) led to premature study‐drug discontinuation, due to a need for antibiotics which were considered a possible risk for additional DDI; the event was considered unrelated to study drugs. All TEAEs were mild (n* *=* *14) or moderate (n* *=* *6; oropharyngeal pain, tooth abscess, increase in levels of alanine aminotransferase [>3 × upper limit of normal (ULN)], and fatigue [3 events]) in severity. The most commonly reported TEAE (≥3 events) was fatigue (8 events in 7 subjects), which was mostly reported (7 of 8 events) in Group 2 between days 14‐23 when simeprevir was coadministered with odalasvir with or without AL‐335. In contrast, during the same days 14‐23 time period in Group 1, only one fatigue event was reported. Since the dosing regimens during this time period were the same, these different fatigue rates across the two groups are considered most likely attributable to chance, although the longer treatment with odalasvir in Group 2 (20 days vs. 10 days in Group 1) cannot be ruled out as a possible etiology to the different rates observed. Importantly, no fatigue event was considered to be clinically significant in this study as they were not associated with other abnormalities, premature study‐drug discontinuation or other medical interventions.

**Table 7:** Summary of treatment‐emergent adverse events (safety population)

n (%)	Group 1 (N* *=* *16)		Group 2 (N* *=* *16)	
	TEAEs, n	Subjects, n (%)	TEAEs, n	Subjects, n (%)
Any TEAE	5	3 (18.8)	15	9 (56.3)
Mild	4	3 (18.8)	10	7 (43.8)
Moderate	1^a^	1 (6.3)	5^b^	4 (25.0)
Severe	0	0	0	0
Serious AE	0	0	0	0
TEAE leading to permanent discontinuation	0	0	1^c^	1 (6.3)
TEAEs				
Diarrhea	1	1 (6.3)	0	0
Abdominal pain	0	0	1	1 (6.3)
Feces, soft	1	1 (6.3)	1	1 (6.3)
Fatigue	1	1 (6.3)	7	6 (37.5)
Muscle spasms	1	1 (6.3)	0	0
Oropharyngeal pain	1	1 (6.3)	0	0
Cytomegalovirus infection	0	0	1	1 (6.3)
Tooth abscess	0	0	1	1 (6.3)
ALT increased	0	0	1	1 (6.3)
AST increased	0	0	1	1 (6.3)
Insomnia	0	0	1	1 (6.3)
Night sweats	0	0	1	1 (6.3)

ALT, alanine aminotransferase; AST, aspartate aminotransferase; TEAE*,* treatment‐emergent adverse event^.^

a Oropharyngeal pain after simeprevir + odalasvir.

b Tooth abscess after odalasvir alone; ALT increase and fatigue (3 cases) after AL‐335 +  simeprevir + odalasvir.

c Tooth abscess, considered to be unrelated to any of the study medications.

TEAEs were more common in Group 2 than in Group 1 (5 TEAEs in 3 subjects in Group 1 and 15 TEAEs in 9 subjects in Group 2), with the major difference being the incidence of fatigue (1 vs. 7 events in Groups 1 and 2, respectively). After excluding this term, the incidence of TEAEs across the two groups was comparable (4 vs. 8 TEAEs in Groups 1 and 2, respectively).

Despite the numeric difference in incidence of TEAEs across the two groups, none of the events in either group appear to have been clinically concerning as evidenced by a lack of severe events or requirement of treatment discontinuation (with the exception of the subject that discontinued study treatment due to a tooth abscess). Only two of the 20 TEAEs reported, oropharyngeal pain and tooth abscess, required concomitant treatment.

One study subject experienced clinically significant laboratory elevations in serum alanine aminotransferase (peak 5.5 × ULN; grade 3) and aspartate aminotransferase (peak 3.8 × ULN; grade 2) from day 26 to day 36, which were considered to be attributable to the onset of a new cytomegalovirus infection (diagnosed by changes in immunoglobulin M and immunoglobulin G values) and not to any of the three study drugs. No other clinically significant changes were observed with respect to laboratory safety parameters (including hematology, blood chemistry, coagulation, and urinalysis parameters), vital signs, physical examination, or ECGs.

## 4 DISCUSSION

The aim of this study was to investigate potential DDIs between AL‐335, odalasvir, and simeprevir in healthy subjects in order to increase our understanding of the pharmacokinetics and safety of the combination, and to guide dose selection of the individual components for the development of a 2‐ or 3‐DAA combination regimen for the treatment of HCV.

Odalasvir and simeprevir, when administered independently and in combination, had a marked effect on AL‐335 and ALS‐022399 exposure but only odalasvir increased ALS‐022227 exposure to a statistically significant extent. The effect on AL‐335 was characterized by a 2‐ to 4‐fold increase in *C*_max_ and AUC_0‐24 h_ which was additive, increasing to 5‐ to 8‐fold when odalasvir and simeprevir were coadministered. This was expected as AL‐335 is a substrate of P‐glycoprotein which is inhibited in vitro by odalasvir and simeprevir. Simeprevir is a clinically confirmed inhibitor of P‐glycoprotein at a dose of 150 mg QD.^15^ The inhibition of intestinal P‐glycoprotein mediated efflux of AL‐335 by odalasvir and simeprevir can contribute to the observed interaction. In contrast, AL‐335 had no impact on the pharmacokinetics of either odalasvir or simeprevir after repeated concomitant administrations, which is consistent with the observation that AL‐335 is a substrate not an inhibitor or inducer.

Odalasvir and simeprevir statistically significantly affected the pharmacokinetics of each other, producing an approximate 1.5‐ to 2‐fold increase in exposure (*C*_max_, *C*_min_, AUC_0‐24 h_) for both drugs. Odalasvir is a weak OATP1B1 substrate in vitro and simeprevir is a clinically confirmed OATP1B1 inhibitor at 150 mg QD. Simeprevir is a P‐glycoprotein substrate and odalasvir is a P‐glycoprotein inhibitor in vitro. These mechanisms may have contributed to the observed increases in odalasvir and simeprevir exposure.

Despite observing higher exposures than expected, the 2‐DAA and 3‐DAA regimens were well tolerated during the short duration of treatment. The only noteworthy TEAE observed was an excess of fatigue events in Group 2 versus Group 1, which does not appear to be clinically concerning (i.e., it did not result in discontinuation of study drugs or otherwise require medical intervention) and is presumed to be related to chance. The only other noteworthy safety finding was the Grade 3 elevation of alanine aminotransferase/aspartate aminotransferase in a subject which occurred concurrent to seroconversion for cytomegalovirus. This is considered likely attributable to the infection (which is commonly associated with transaminitis) rather than being drug‐induced. Although these observations are not considered attributable to exposure to the study drugs, additional surveillance for these TEAEs will nevertheless continue to occur in future clinical trials in HCV‐infected patients, where assessment of safety parameters such as TEAEs (particularly fatigue) and liver function tests is standard practice.

An important strength of the study was its design, which allowed for the assessment of complex 2‐ and 3‐way drug interactions between AL‐335, odalasvir, and simeprevir. A limitation of the study is that we were unable to evaluate any potential impact on the active entity of AL‐335, nucleoside triphosphate, as this is only present intracellularly. Thus, how any change in plasma kinetics of the AL‐335 analytes may translate to the modulation of liver nucleoside triphosphate remains unknown. The effect of the DDI on viral‐load reduction can only be evaluated when this DAA combination is evaluated in patients. In addition, differences in metabolism and transporter expression between healthy volunteers and HCV‐infected patients may limit extrapolation of results.^21^ Another limitation is that the effect of simeprevir on the pharmacokinetics of odalasvir was estimated without taking into account odalasvir accumulation over time; therefore, the data presented assume steady‐state of odalasvir. This can be corrected for by comparing data between Groups 1 and 2 after 7 days of treatment with odalasvir. When the difference in odalasvir treatment duration is accounted for, the effect of simeprevir on odalasvir is reduced with a 10‐15% C_max_ increase and 12‐19% AUC increase until day 17, compared with a 25% and 50% increase, respectively, at day 20 without accounting for this difference.

The data from this study were used to guide the initial dose selection in Phase IIa and Phase IIb studies. The Phase IIa study, AL‐335‐604 (NCT02569710), is an ongoing study assessing the safety, pharmacokinetics, and efficacy of a 2‐ and 3‐DAA regimen comprising AL‐335 in combination with odalasvir with or without simeprevir in HCV‐infected patients.^22^ The Phase IIb study, OMEGA‐1 (ClinicalTrials.gov ID: NCT02765490), is evaluating AL‐335, odalasvir, and simeprevir for 6 and 8 weeks' treatment duration in HCV genotype 1‐, 2‐, 4‐, 5‐, and 6‐infected patients without cirrhosis.^23^

In conclusion, in this study, combinations of AL‐335, odalasvir, and simeprevir were found to be well tolerated in healthy subjects despite the observed higher exposures of all drugs, in particular AL‐335. These findings have helped to refine the dose selection of each component to establish a regimen for evaluation in Phase II studies in patients with chronic HCV infection.

## AUTHOR CONTRIBUTIONS

All authors participated in the interpretation, critical review, and revision of the final manuscript. TNK, MWM, JV, M‐CH, AP, and JF participated in the design and execution of the trial. CW participated in the execution, oversight, and analysis of the trial and its data. GP, LV, CD, JH, DA, DBS, and SMC participated in the design and interpretation of the trial. QZ oversaw the bioanalysis of the plasma samples from this study and reviewed the bioanalytical and pharmacokinetic data generated.

## DISCLOSURE

TNK, CW, JV, QZ, DBS, SMC, and JF are employees of Alios BioPharma, Inc., part of the Janssen Pharmaceutical companies, and shareholders of Johnson & Johnson. MWM was an employee of Alios BioPharma Inc., part of the Janssen Pharmaceutical companies at the time of the study. M‐CH, GP, LV, CD, and AP are employees of Biotrial and have no competing interests. JH is an employee of Achillion Pharmaceuticals, Inc. and has no competing interests. DA was an employee of Achillion Pharmaceuticals, Inc. at the time of the study, and has no competing interests.
