# Supplementary material for: Pharmacokinetics, safety, and tolerability of the 2‐ and 3‐direct‐acting antiviral combination of AL‐335, odalasvir, and simeprevir in healthy subjects
Source: Pharmacol Res Perspect. 2018 Apr 30;6(3):e00395. doi: 10.1002/prp2.395 (PMC5927802; doi:10.1002/prp2.395)
Supplement: Supplementary file 1 [file PRP2-6-e00395-s001.docx]

**Supplementary Appendix**

**Inclusion and exclusion criteria**

Subjects were healthy volunteers aged 18–60 years with a body mass index (BMI) of 18–32 kg/m^2^ (minimum weight 50 kg and ≤25% of subjects were permitted to have a BMI ≥30 kg/m^2^). Subjects were in good health as deemed by the investigator, based on the findings of a medical evaluation including medical history, physical examination, laboratory tests, and electrocardiogram (ECG). Female subjects were eligible to participate if they were of non-childbearing potential or postmenopausal. Male subjects had to be surgically sterile or practicing specific forms of birth control until 6 months after the end of the study. Male subjects had to agree to refrain from sperm donation from check-in through 6 months after dosing. Subjects were asked to avoid prolonged sun exposure and to use precautions to help protect against potential phototoxicity due to simeprevir.

Exclusion criteria included clinically significant cardiovascular, respiratory, renal, gastrointestinal, hematological, neurological, thyroid, or any other medical illness or psychiatric disorder, as determined by the Investigator and/or Sponsor’s Medical Monitor; any condition that, in the opinion of the investigator, would have compromised the study’s objectives or the well-being of the subject or prevented the subject from meeting the study requirements; participation in an investigational drug trial or having received an investigational vaccine within 30 days or 5 half-lives (whichever was longer) prior to study medication; clinically significant cardiac disease including abnormal ECG findings; particularly, a history or family history of prolonged QT syndrome (e.g. torsade de pointes) or sudden cardiac death; ECG with PR >200 ms, QRS >120 ms, QTcF >450 ms, as assessed by centrally read 12‑lead ECG at the Screening Visit; clinically significant anemia; abnormal biochemistry or hematology laboratory results obtained at screening; abnormal heart rate, respiratory rate, temperature or blood pressure values outside of the normal range; active infection; history of regular alcohol intake or use of tobacco/nicotine-containing products; East Asian ancestry (due to pharmacokinetic differences with simeprevir); a positive pre-study drug screen. The use of concomitant medications, including prescription, over-the-counter medications, herbal medications, inducers or inhibitors of cytochrome P450 enzymes or drug transporters (including P‑glycoprotein) within 14 days prior to the first dose of study medication was excluded, unless approved by the Sponsor’s Medical Monitor. Occasional use of acetaminophen was permitted. Subjects were also excluded if they had been exposed to more than four new investigational entities within 12 months prior to the first dosing day or if they were hypersensitive to the active substances or to any of the excipients of AL-335, odalasvir, or simeprevir. Women who were pregnant or nursing or contemplating pregnancy were excluded, as were men whose female partners were pregnant or contemplating pregnancy. Subjects previously infected with hepatitis C virus and having achieved sustained virologic response on treatment (no detectable hepatitis C virus RNA 6 months post-treatment) were eligible.
